# Current status of the sterile insect technique for the suppression of mosquito populations on a global scale

**DOI:** 10.1186/s40249-024-01242-z

**Published:** 2024-09-26

**Authors:** Jérémy Bouyer

**Affiliations:** 1 Insect Pest Control Laboratory, Joint FAO/IAEA Centre of Nuclear Techniques in Food and Agriculture, IAEA, Vienna, Austria; 2 ASTRE, CIRAD, 34398 Montpellier, France; 3 ASTRE, Cirad, INRAE, Univ. Montpellier, Plateforme Technologique CYROI, Sainte-Clotilde, La Réunion France

**Keywords:** Irradiation, Autocidal control, Vector control, Integrated vector management, Integrated pest management, Dengue, Arbovirus, *Aedes*

## Abstract

**Background:**

The World Health Organization (WHO) has emphasized the urgent need for alternative strategies to chemical insecticides for controlling mosquito populations, particularly the invasive *Aedes* species, which are known vectors of arboviruses. Among these alternative approaches, the sterile insect technique (SIT) is experiencing rapid development, with numerous pilot trials being conducted worldwide.

**Main text:**

This review aims to elucidate the principles of SIT and highlight the significant recent advancements that have facilitated its scalability. I also employ a phased conditional approach to categorize the progression of 39 projects, drawing on peer reviewed studies, press releases and direct communication with project managers. This review indicates that a substantial number of projects illustrate the efficacy of SIT in suppressing *Aedes* populations, with one project even demonstrating a reduction in dengue incidence. I offer several recommendations to mitigate potential failures and address the challenges of compensation and overcompensation when implementing SIT field trials. Furthermore, I examine the potential implications of male mating harassment on the effectiveness of SIT in reducing disease transmission.

**Conclusions:**

This comprehensive assessment underscores the promise of SIT as a viable strategy for mosquito control. The insights gained from these trials not only contribute to the understanding of SIT’s effectiveness but also highlight the importance of careful project management and ecological considerations in the pursuit of public health objectives.

**Graphical Abstract:**

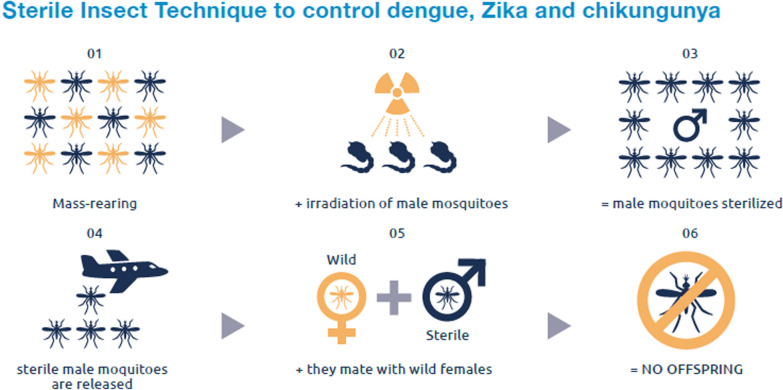

**Supplementary Information:**

The online version contains supplementary material available at 10.1186/s40249-024-01242-z.

## Background

Vector-borne diseases represent a significant global health challenge, accounting for 17% of all infectious diseases and resulting in more than one million deaths annually, as reported by the World Health Organization (WHO) [1]. Among these diseases, malaria, transmitted by *Anopheles* mosquitoes and diseases caused by arboviruses such as dengue, chikungunya, yellow fever and Zika, transmitted by *Aedes* mosquitoes, are of particular concern. Growing awareness about the toxicity of chemical insecticides to both living organisms and ecosystems has prompted many countries to limit the number of approved molecules. Moreover, the increasing resistance to pyrethroids—the most commonly used class of insecticides—poses a looming risk of their potential withdrawal in the near future. In response to these challenges, the WHO has advocated for the development of alternative vector control methods targeting mosquitoes [1].

In recent years, genetic control technologies have gained traction globally as viable alternatives to chemical insecticides, acknowledging their detrimental effects on ecosystems and human health. Notably, the sterile insect technique (SIT) has experienced renewed interest as a method for controlling *Aedes* mosquitoes [2], particularly following the Zika virus outbreaks in the Americas and the rising incidence of dengue fever. The WHO and the International Atomic Energy Agency (IAEA) have provided guidelines for testing SIT against *Aedes* mosquitoes [3], which serve as the foundation for upcoming trials in the Pacific region. This raises a pertinent question: what is the status of other ongoing trials worldwide?

### New developments in the SIT approach against *Aedes* mosquitoes

**Fig. 1:**
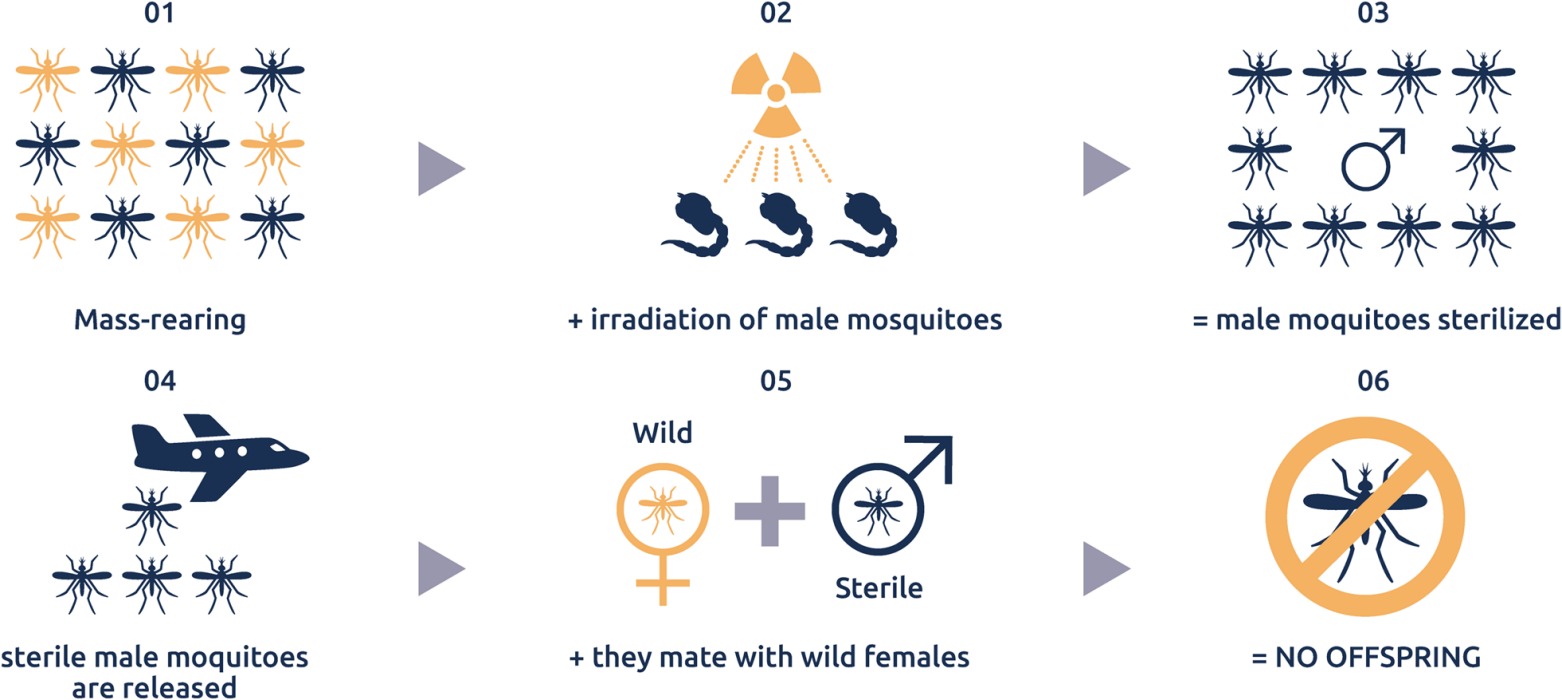
Principle of the sterile insect technique against mosquitoes (Source: [3])

The SIT operates on the principle of releasing irradiated sterile male mosquitoes into a designated, where they mate with wild females, resulting in the production of no viable offspring (Fig. 1). This species-specific, environmentally friendly autocidal method has a long history of successful large-scale implementation against various insect pests since the 1950s, and it is exempt from genetically modified organism (GMO) regulations [4]. In this paper, I did not consider other ways to sterilize the males like chemical treatment, even if a very successful project was recently reported [5]. Also, several other technologies with similar principles of action are not considered here. These include:

- The incompatible insect technique (IIT), where the induced sterility is conditional to the status of the target population and that can lead to resistance [6];
- The RIDL (release of insects carrying a dominant lethal gene) where the released males are not sterile but result in non-viable or male-biased progeny [7];
- PgSIT (precision guided sterile males), which is based on the crossing of two transgenic lines to produce sterile males and has not been tested in the field yet [8];
- Gene drive, which may allow in the future to drive maleness in target populations but which has also not been tested in the field yet [9].

Mutations resulting from exposure to radiation are inherently random, they are different in each released insect. This randomness inherently limits the potential for the target population to develop resistance, which stands as one of the key advantages of this technology. Furthermore, SIT can be integrated with IIT. In this combination, complete male sterility is induced by *Wolbachia* (which causes cytoplasmic incompatibility) and is conditional upon mating with wild females, while the sterility of residual females is induced by radiation [10]. By sterilizing females released accidentally, the risk of unintended population replacement—often observed when IIT is employed alone—is mitigated. Additionally, the reduced irradiation dose enhances the competitiveness of sterile males. Another innovative variant, known as boosted SIT, utilizes sterile males not only to induce sterility but also to serve as carriers of biocides targeted at females and their larval habitats [11]. This approach can be particularly effective at the onset of a suppression effort, followed of standard SIT. Its efficiency has recently been demonstrated at a small scale using pyriproxyfen as a biocide in both France and Spain [12]. Pyriproxyfen is a larvicide preventing the metamorphosis of larvae and nymphae into adults, which has previously been used in auto-dissemination stations against *Aedes* species [13].

- The SIT framework for combating mosquito populations encompasses several critical components: strain development, mass rearing, sex separation, sterilization, quality control, handling, transportation and the release of the sterile males. Recent advancements in this area include: The development of more affordable larval diets utilizing insect proteins [14];
- Enhanced feeding protocols along with cost-effective mass-rearing racks and adult cages [15, 16];
- The introduction of automatic sex-sorters that improve the speed of sex separation by up to 17 times compared to traditional manual Fay-Morlan sorters [17];
- Greater understanding of the factors influencing the quality of sterile males when applying pupae irradiation [18], along with the establishment of mass-irradiation protocols for adult mosquitoes [19];
- Standardization of a flight test device that facilitates rapid quality control of sterile males [20], as well as guidelines for assessing their performance in the field through mark-release-recapture (MRR) experiments;
- Development of handling and transportation protocols for chilled adult mosquitoes [21];

Implementation of aerial release systems for sterile male mosquitoes using drones [22]. Recent advancements have contributed to a reduction in the production costs of sterile male mosquitoes, although a perfect genetic sexing system remains unavailable. Ongoing efforts are focused on developing such systems [23, 24] and may allow a further drastic reduction of the cost of SIT against mosquitoes in the near future.

### The phase conditional approach to testing SIT against *Aedes* mosquitoes

The evaluation of SIT as a new control tool will ultimately rely on the phase conditional approach (PCA) proposed by the WHO Vector Control Advisory group (VCAG) [3]. However, a more tailored guideline [25] for mosquito SIT has been introduced, motivated by two primary factors (see Fig. 2 of [26]). First, SIT cannot function as a standalone solution; it must be integrated with other control methods due to its inverse density-dependent properties, which differ from most products evaluated by VCAG. Second, for SIT to be effective, it needs to be implemented area-wide, leading to specific testing requirements that draw from lessons learned in the operational deployment against other pest species, particularly fruit flies and tsetse flies.

**Fig. 2:**
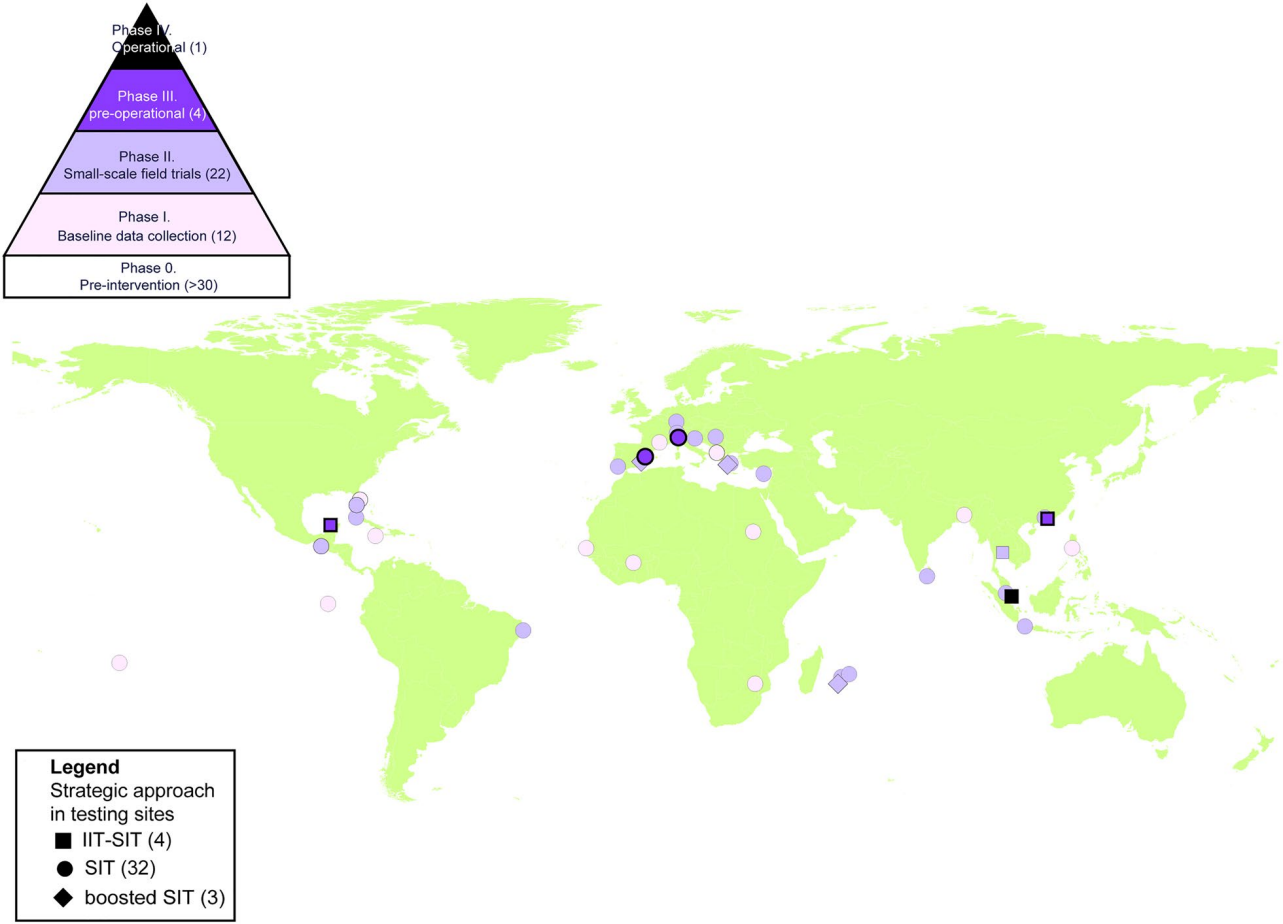
Global distribution of 39 projects releasing irradiated sterile males. *IIT* incompatible insect technique; *SIT* sterile insect technique. Source of the world map: world administrative boundaries from opendatasoft

The proposed PCA for mosquito SIT serves as a valuable resource for decision makers and research teams in preparing and executing SIT strategies against mosquitoes [25]. Progression to the next phase is contingent upon the completion of most activities in the preceding phase, effectively minimizing the risk of financial losses, as each subsequent phase can incur costs up to ten times greater than its predecessor. It is essential to identify relevant and committed stakeholders for each phase, ensuring they are well informed and adequately trained throughout the decision-making process. Capacity building and expertise tailored to each phase is crucial for developing the technical skills. Additionally, external review meetings with international experts should be convened at the conclusion of each phase to provide recommendations on whether to proceed to the next phase.

During phase 0 (pre-intervention), it is essential to secure governmental commitment to introduce SIT in its integrated vector control strategy (IVM) must be confirmed. This includes not only political backing but also financial support to ensure the program's sustainability. A comprehensive program for mosquito control should be established, featuring dedicated personnel who are adequately trained and equipped, along with a designated budget. Additionally, there should be an overarching national or regional strategy that focuses on integrated mosquito control.

This may be evaluated through the framework for a national vector control needs assessment proposed by the WHO [1]. This framework has been tailored to encompass specific details pertinent to SIT, such as the availability of irradiation facilities. At this stage, it is also crucial to gather all existing entomological and epidemiological data on mosquito-borne diseases from national or regional monitoring systems.

During phase I (baseline data collection), training in mosquito taxonomy, field data collection, data storage and analysis, and field monitoring must be provided. Identifying suitable field sites for future testing is also crucial, as the initiation of a baseline data collection. This data should encompass entomological aspects, including the spatio-temporal characterization of the wild populations, as well as epidemiological, socio-economic and environmental data. Establishing an insectary is essential, where a local mosquito strain can be colonized. Routine colony rearing and maintenance these colonies must be implemented, alongside studies to characterize life history traits, compatibility assessments and semi-field competitiveness studies. Securing irradiation capacity is another vital step, which includes ensuring access to an irradiator, implementing dosimetry, and gathering dose–response information relevant to the strain and irradiator being utilized. It is also important to train staff on standard operating procedures for various laboratory processes, including rearing, sex separation, irradiation, handling, marking, transportation and releasing of sterile male mosquitoes. Finally, a MRR should be conducted to evaluate the survival, dispersal and field competitiveness of sterile males in open field conditions.

During phase II (small-scale field trials), it is essential to develop a comprehensive communication plan aimed at gaining the support of beneficiary communities. Identifying and testing complementary suppression methods to be integrated alongside SIT within an integrated vector management framework is crucial. All necessary authorizations for the release of sterile males must be secured, including import permits if the production of sterile males is outsourced. If deemed necessary, a risk assessment analysis will be conducted at this stage. The execution of a small-scale field trial is vital, encompassing the entire process from rearing to the monitoring of entomological and potentially epidemiological indicators. This will necessitate an adequate colony size or the outsourcing of sterile male production. The results obtained from this field trial should be disseminated, and a cost-benefit analysis for a potential operational program should be initiated.

Phase III (pre-operational), often regarded as the most challenging step, is initiated only if the country opts to incorporate SIT to its large-scale integrated vector control strategy. At this juncture, a mass-rearing and mass-sterilization capacity will be established, coupled with intensified community outreach initiatives in targeted areas, expanding upon the efforts made in phase II. During this phase, comprehensive strategies for storage, handling, transport, release and monitoring strategies for upscaled application will be developed to support the upscaling of operations. Ongoing quality control measured will be implemented to ensure the integrity of the process. Additionally, a management plan and structure will be put in place to facilitate the operational application of SIT. The data generated from these larger-scale operations will be utilized to evaluate the cost-efficiency of the technology and to inform the development of a market model.

In phase IV, a large-scale SIT operational program will be implemented, from mass-rearing to aerial releases under an adaptive management framework. Monitoring activities will be conducted in targeted areas, albeit with a lower resolution to enable costs savings. Feedback mechanisms will be established between field, mass-rearing facilities and management teams to enhance communication and efficiency. Continuous analysis of field results, along with the assessment of the impact of releases on entomological and epidemiological indicators, will be undertaken. This ongoing evaluation will inform strategies for improving the efficiency and cost-effectiveness of the IPM strategy, which includes the SIT component. Problem-solving research and routine external reviews will be integral to this process, ensuring that the program remains responsive to emerging challenges and opportunities.

### Current SIT projects targeting mosquitoes on a global scale

I used the PCA presented above to categorize the stage of 39 ongoing projects in 2024 (Fig. 2, Suppl. Table 1). Most of these initiatives focus on *Aedes* species, with 18 projects specifically targeting *Ae. albopictus*, 18 aimed at *Ae. aegypti* and 1 addressing both species. In contrast, only two projects are directed towards *Anopheles arabiensis*.

A significant number of projects that were previously in phase I have either ceased operations, failed to provide updates, or progressed to phase II, resulting in the initiation of 11 new projects. Notably, only three projects have advanced to phase III: the IIT-SIT initiative in Mexico, and SIT programs in Spain and Italy. Additionally, one project has reached phase IV, specifically the IIT-SIT project in Singapore (Table 1). Several ongoing phase II projects, including those in Brazil, France, Greece, and Serbia are actively seeking funding to facilitate their transition to phase III. The stakeholders involved in these projects comprise a mix of government agencies, research groups or private companies. The notable growth of SIT trials in Europe is particularly striking, given their primary objective of preventing outbreaks of arboviruses that are not typically endemic to the region.

**Table 1:** Dynamics of sterile insect technique projects against mosquitoes along the phase conditional approach

Phase	I	II	III	IV	Total
2019	21	11	2	0	34
2024	12	22	4	1	39
Increase rate	− 43%	100%	50%	100%	NA

*NA* not applicable

The countries presented in 2019 and 2024 are not identical: no updated data were available for some of them whereas new countries initiated trials during this period

The commercialisation of sterile male insects by Centro Agricoltura Ambiente “G. Nicoli” (CAA), an IAEA collaborating centre in Italy, alongside the establishment of mass transportation protocols, has enabled the execution of MRR experiments and phase II pilot trials across several European countries, including Albania, Croatia, France, Greece, Montenegro, Portugal, and Serbia. This regional production initiative has significantly bolstered the development of SIT in Europe through two IAEA Technical Cooperation regional projects (RER5022 and RER5026). In some instances, countries such as Croatia and Serbia opted to utilize the Italian strain of *Ae. albopictus* instead of their own local strains; however, most countries requested CAA to mass-rear their local strains. In Cyprus, sterile male *Ae. aegypti* were produced by the FAO-IAEA Insect Pest Control Laboratory in Austria in response to a new invasion of the island by this species. This initiative marks the first effort aimed at the elimination of the target population (Suppl. Table 1). For the phase II pilot trials, the average size of the target areas was 38 hectares, although there was considerable variation, ranging from 1.2 to 230 hectares. The average release density was approximately 15,000 sterile males per hectare per week, with significant fluctuations observed. The release density varied from as few as 353 sterile males per hectare per week in the boosted SIT project on Reunion Island to as high as 166,666 sterile males per hectare per week in the IIT-SIT project in Guangzhou. It is important to note that there was no direct correlation between release density and suppression rates, which ranged from 0 to 100% (see details in Suppl. Table 1). A significant characteristic of successful projects is their ability to garner public support, which is essential for the seamless progression of SIT projects. This support has largely been attributed to well-structured communication strategies that effectively engage diverse audiences. Additionally, the minimal presence of substantial ethical concerns related to this technology has contributed to its widespread acceptance across various cultural contexts. This combination of effective communication and cultural receptivity played a pivotal role in advancing SIT initiatives.

## Conclusions

The ongoing evaluation of SIT projects indicates that the effectiveness of SIT against mosquitoes is contingent upon adherence to specific testing and operational conditions [3]. Notably, Singapore's project, which has progressed to phase IV, serves as a model due to its comprehensive approach. This initiative has successfully implemented a PCA, demonstrated significant entomological [27] and epidemiological impacts [28], and showcased cost-efficiency [29], all while maintaining robust public communication to garner support. Consequently, the integration of the IIT-SIT into Singapore’s national dengue control strategy, as managed by the National Environment Agency, presents a valuable reference for other nations in earlier phases, potentially expediting their SIT development efforts. It must be noted that this project is an IIT-SIT combination, not a standard SIT one. The logic behind combining *Wolbachia*-induced sterility with SIT lies in minimizing radiation exposure, thereby preserving a good competitiveness of sterile males. However, most of the suppression effect can be attributed to SIT [30] and advancements in mass-irradiation protocols for adult mosquitoes may enable countries engaged in standard SIT to achieve similar or even superior competitiveness [31]. Currently, most countries are still using pupae irradiation, with the exceptions of Brazil, China and Spain. This improvement is crucial, as previous quality losses were primarily linked to anoxia resulting from high density pupae irradiation [32, 33]. The relatively lower transition of countries from phase II to phase III can be attributed to the substantial increase of costs and the necessity for investments in local production capacity. Mass-production in phase III can pose significant challenges; factors that were manageable at smaller scales, such as producing 100,000–200,000 sterile males weekly, can become bottlenecks when production scales exceed one million sterile males weekly. One major cost reduction might come from the development of a fully operational genetic sexing strain [23].

When embarking on mosquito SIT trials, it is essential for countries to identify complementary suppression methods to initially reduce mosquito populations. For instance, public education aimed at reducing larval habitats can be beneficial. Moreover, pilot tests should be conducted in isolated areas or include a buffer of at least 200 m with release of sterile males to reduce the immigration of fertile females from surrounding areas. Target areas should ideally cover a minimum of 50 hectares. In scenarios where induced sterility is at or below 50%, minimal effects on the target mosquito populations may be observed, often due to the existence of compensation and overcompensation in larval mortality [34].

This remarkable resilience observed in *Aedes* mosquitoes prompts specific recommendations for the implementation of SIT: (i) competitiveness must be assessed in the field and should exceed 0.2 prior to initiating suppression trial; (ii) the release density must be sufficient to achieve an induced sterility rate of at least 0.7 to prevent density-dependant compensation. An alternative approach is to utilize boosted SIT with pyriproxyfen [11] at the commencement of SIT trials to achieve an initial reduction, as this biocide aids in preventing compensation and offers partial protection against the immigration of fertile females. In some trials, an unexpected reverse phenomenon has been noted, where the reduction rate surpasses induced sterility, a situation typically deemed unlikely under standard SIT mechanisms (which rely on egg and early larval mortality). This occurrence was particularly pronounced in China, where a 40% suppression of female mosquitoes, coupled with an 80% decrease in biting rates, was recorded despite minimal induced sterility. This effect was attributed to reduced female survival and feeding success due to male mating harassment [35]. Given that increased female mortality and decreased host-vector contact can immediately lower disease transmission, it will be crucial in future SIT trials to monitor key entomological indicators, including female age, biting rates, in addition to their overall density, alongside egg counts.

## Supplementary Information

Additional file 1 (DOCX 44 kb)

## Data Availability

All data used in this review are presented in Supplementary Table 1.
