# Supplementary material for: Current status of the sterile insect technique for the suppression of mosquito populations on a global scale
Source: Infect Dis Poverty. 2024 Sep 26;13:68. doi: 10.1186/s40249-024-01242-z (PMC11426227; doi:10.1186/s40249-024-01242-z)
Supplement: Supplementary file 1 — Additional file 1 (DOCX 44 kb) [file 40249_2024_1242_MOESM1_ESM.docx]

**Supplementary Material**

**Supplementary Table 1:** List of ongoing SIT projects against mosquitoes. Updated from Tables 4 and 5 from [1]. This list is not exhaustive: all projects where no updates were found online in February 2024 were removed from the list.

| **Country** | **City** | **Approach** | **Size of release area (ha)** | **Inhabitants in the release area** | **Average release density (/ha/w)** | **Current status & perspectives** | **Species** | **Phase in**  **2024** | **Source** |
| --- | --- | --- | --- | --- | --- | --- | --- | --- | --- |
| Albania | Tirana | SIT | 12 | NA | NA | MRR in 2018 completed with sterile males from the local strain produced in Italy (CAA). Development of local production of sterile males ongoing. | *Ae. albopictus* | I | [2] |
| Bangladesh | Dhaka | SIT | NA | NA | NA | Evaluation of mass-rearing and irradiation procedures in the lab. | *Ae. aegypti* | I | [3] |
| Brazil | Recife | SIT | 56 | 18300 | 5000 | MRR in Carnaiba in 2018 to test drone release. 19% suppression in the wild mosquito population in Recife. Project ongoing to upscale and measure epidemiological impact on Fernando de Noronha island. | *Ae. aegypti* | II | <https://www.iaea.org/newscenter/news/nuclear-science-to-control-mosquitoes-generate-clean-energy-a-key-focus-of-director-general-grossis-visit-to-brazil>  [4, 5] |
| Burkina Faso | Bobo Dioulasso | SIT | NA | NA | NA | Baseline data collection for 3 years. Building and equipment of a mass-rearing facility. | *Ae. aegypti* | I | R. Dabire, pers. com. |
| China | Guangzhou | SIT | 1.2 | 4750 | 166666 | No induced sterility but 40% reduction of female density in BG traps and 80% in Human Landing Catch due to male mating harassment. | *Ae. albopictus* | II | [6] |
| China | Guangzhou | IIT / SIT | 35 | 2215 | 100000 | Field pilot trial completed (>99% suppression of females) and pre-operational trials ongoing for cost reduction, with commercial activity by Guangzhou Wolbaki Biotech Co. Ltd. | *Ae. albopictus* | III | [7] |
| Croatia | Premantura | SIT | 30 | NA | 2000 | Field pilot completed with sterile males from an Italian strain produced in Italy (CAA). 60% induced sterility and 45% suppression of adults. | *Ae. albopictus* | II | <https://www.rfi.fr/en/science-environment/20230720-croatia-targets-latest-climate-change-threat-mosquitoes>  G. Vignjević, pers. com. |
| Cuba | La Habana | SIT | 50 | NA | 1270 | 100% suppression of egg densities in the pilot area. RTC planned in La Habana to measure epidemiological impact. | *Ae. aegypti* | II | [8]  [9] |
| Cyprus | Kiti | SIT | 50 | NA | 2000 | Elimination trial ongoing. Sterile males produced by FAO-IAEA, Austria. No information available on the results. | *Ae. aegypti* | II | [10] https://www.iaea.org/newscenter/pressreleases/nuclear-technique-used-in-europe-for-first-time-to-battle-yellow-fever-mosquito-found-in-cyprus |
| Ecuador | Galapagos | SIT | NA | NA | NA | Small insectary, rearing local strain, irradiation capacity, trapping & monitoring field sites in progress. | *Ae. aegypti* | I | https://www.geneconvenevi.org/articles/galapagos-to-receive-male-mosquitoes-for-vector-control-in-ecuador/ |
| France | French Polynesia | SIT | NA | NA | NA | BLDC. Building of mass-rearing facility, purchase of irradiator. Plan of a pilot trial to measure entomological and epidemiological impacts. | *Ae. aegypti* | I | [11] |
| France | Montpellier | SIT | 10 | NA | NA | MRR to test drone release of sterile males with sterile males from a local strain produced in Italy (CAA). Perspective of upscale by Terratis. | *Ae. albopictus* | I | <https://www.eid-med.org/projet-tis/>  https://terratis.fr/ |
| France | Reunion Island | SIT | 32 | NA | 3000 | Pilot trial completed, 50% induced sterility. Shift to boosted SIT (phase 3) planned in 2024. | *Ae. albopictus* | II | <https://www.ird.fr/la-technique-de-linsecte-sterile-reduit-de-50-la-fertilite-des-moustiques-aedes-albopictus-duparc>  [12, 13] |
| France | Reunion Island | Boosted SIT | 10 | 171 households | 353 | Pilot trial completed, 90% reduction of the target species, 60% reduction of *Ae. albopictus*. Phase 3 planned in 2024. Perspective of upscale by MoSITouch. | *Ae. aegypti* | II | <https://www.cirad.fr/espace-presse/communiques-de-presse/2021/tis-renforcee-moustique-reunion>  [14]  https://www.mositouch.com/ |
| Germany | Heidelberg & Freiburg | SIT | 10 | NA | 1000-2300 | Field pilot completed with sterile males from a local strain produced in Italy (CAA). 56-82% induced sterility. No information available on a follow-up. | *Ae. albopictus* | II | [15] |
| Greece | Vavrona (Athens) | SIT | 10 | NA | 3000 | Field pilot completed with sterile males from a local strain produced in Italy (CAA). 78% reduction in egg density. Shift to a phase 2 trial of boosted SIT in the same site (ongoing) / development of local production of sterile males ongoing. | *Ae. albopictus* | II | [16] https://www.mosquitosit.gr/ |
| Greece | Vavrona (Athens) | SIT | 10 | NA | 3000 | Intermitent releases (beginning and end of the mosquito season). >95% reduction of adult density measured with Human Landing Catch | *Ae. albopictus* | II | 1. Michaelakis, pers. com. |
| Indonesia | Pasar Jumat-Lebak Bulus (Jakarta) | SIT | 25 | NA | NA | 50% induced sterility. Extension of the trials ongoing. | *Ae. aegypti* | II | [17] |
| Italy | Caselline, Boschi, Budrio, Santamonica | SIT | 80 | NA | 1600 | Field pilot completed in 2013. 70–80% induced sterility and suppression of egg densities. | *Ae. albopictus* | II | [18] |
| Italy | Bologna | SIT | 70-100 | NA | 2081-3265 | 34-48% induced sterility and 47-56% egg reduction from 2021-2023. Pre-operational trials ongoing for cost reduction, with commercial activity by Centro Agricoltura Ambiente “G.Nicoli” (CAA). | *Ae. albopictus* | III | [19]  Information provided by CAA. |
| Jamaica | Kingston (St. Catherine Parish) | SIT | NA | NA | NA | Medium scale production, trapping & monitoring field sites, MRR. No update on the field trial available. | *Ae. aegypti & Ae. albopictus* | I | https://jis.gov.jm/jamaica-to-undertake-mosquito-sterilisation-pilot-project/ |
| Malaysia | Melaka state | SIT | 4 | 16000 | NA | MRR conducted. Extension of the trials ongoing. | *Ae. aegypti* | II | [20, 21] |
| Mauritius | Panchvati | SIT | 3 | NA | 20000 | 55.7 % suppression of egg densities and 63.6% suppression of adult females. New pilot trial ongoing in an urban area (Ministry of Health). | *Ae. albopictus* | II | [22] |
| Mexico | Tapachula | SIT | 24 | 697 | 6000 | BLDC. MRR conducted to assess drone release of sterile males. No available report on the suppression trial. No information available on a follow-up. | *Ae. aegypti* | II | [23, 24] |
| Mexico | Merida | IIT / SIT | 50 | 1241 | 4000 | Pilot trial show suppression of egg hatch by 76.5 -91.9%, indoor females by 47.7-90.9% and outdoor females by 50.0–75.2%. Ongoing USAID project will test the impact on disease transmission and cost effectiveness at scale for transition to phase IV. | *Ae. aegypti* | III | [25]  <https://divportal.usaid.gov/s/project/a0g3d000000cNZAAA2/testing-a-combined-sitiit-approach-to-control-mosquitoborne-diseases-at-scale?utm_medium=email&utm_source=govdelivery> |
| Philippines | Old Balara | SIT | NA | NA | NA | Building of mass-rearing facility, purchase of irradiator. | *Ae. aegypti* | I | [26] |
| Portugal | Faro | SIT | NA | 1946 | 4000 | Field pilot completed with sterile males from the local strain produced in Italy (CAA). No result published yet. | *Ae. albopictus* | II | H. C. Osório, pers. com. |
| Senegal | Dakar | SIT | NA | NA | NA | MRR in Dakar using sterile males produced in Austria and transported as chilled irradiated adults in insulated boxes (FAO-IAEA Insect Pest Control Laboratory). | *Ae. aegypti* | I | [27]  Gueye Fall & T. Bakhoum, pers.com. |
| Serbia | Novi Sad | SIT | 17.2 | 3000-4000 | 4444 | Field pilot completed with sterile males from an Italian strain produced in Italy (CAA). 51.5% induced sterility and 64% suppression of egg density. Perspectives of upscale through a national IAEA TC project and the private company BIODRON 369. | *Ae. albopictus* | II | <https://www.srbatom.gov.rs/srbatomm/pedeset-hiljada-sterilnih-muzjaka-tigrastog-komarca-pusteno-u-novom-sadu-u-okviru-projekta-za-kontrolu-stetocina-na-ekoloski-prihvatljiv-i-odrziv-nacin/?lang=en>   1. I. Cupina, pers. com. |
| Singapore | Yishun, Tampines, Bukit Batok and Choa Chu Kang | IIT / SIT | 1033 | 607 872 | NA | Suppression of adult females by 92.7% and 98.3% in two pilot sites. Epidemiological trial demonstrated an average reduction in dengue incidence rate of 56·88% overall (95% CI 51·88–58·46) at an average coverage of 34·49%; 65·81% (64·24–67·26) at a 68·07% coverage and up to 71·01% (163 of 230, 69·47–72·41) in Yishun, at a 72·79% coverage. Operated by the National Environment Agency within the country’s integrated vector control management strategy. | *Ae. aegypti* | IV | [28, 29] |
| South Africa | KwaZulu / Natal | SIT | 5 | NA | 5000 | BLDC, operational research, insectary with irradiation capacity, communication campaign, MRR | *An. arabiensis* | I | [30] |
| Spain | La Vilavella & Polinyà de Xuquer | Boosted SIT | 58 | NA | 2620 - 2712 | Suppression of adults by 89 to 98%. | *Ae. albopictus* | II | [14] |
| Spain | Valencian Community | SIT | 177 | NA | 2250 | 19.5 -31.7% induced sterility, concurrent to a 70–80% suppression of adult females in a 45ha pilot area. Pre-operational trials and R&D activities for cost reduction ongoing by Generalitat Valenciana and Grupo Tragsa. | *Ae. albopictus* | III | [31, 32] |
| Sri Lanka | Colombo | SIT | 30 | NA | 3300 | 95.5% suppression of adult females. Extension of the trials ongoing. | *Ae. albopictus* | II | Prof. Menaka D. Hapugoda, pers com.  [33] |
| Sudan | Dongola | SIT | NA | NA | NA | MRR in 2016. Dose-response curve, development of mass-rearing. Field trial cancelled. | *An. arabiensis* | I | [34, 35] |
| Switzerland | Ticino | SIT | 45 | NA | 3000 | Field pilot completed with sterile males from the local strain produced in Italy (CAA). 18% induced sterility and 66.7% reduction in adult females density. | *Ae. albopictus* | II | [36] |
| Thailand | Plaeng Yao District | IIT / SIT | 5 | NA | 5000 | 84% induced sterility and 97.3% suppression of the mean number of females per household. Plan for a RCT to measure epidemiological impact presented to WHO-VCAG but no information available on a follow-up. | *Ae. aegypti* | II | [37] |
| USA | Captiva island, Florida | SIT | 230 | 379 | 5000 | Suppression > 95% of adults. Shift to Fort Myer because of a cyclone. | *Ae. aegypti* | II | [38] |
| USA | St. Augustine, Florida | SIT | NA | NA | NA | Dose-response curve conducted. Pilot site identified. | *Ae. aegypti* | I | [39] |

**BLDC** = baseline data collection conducted. **SIT**= Sterile insect technique; **IIT**= Incompatible insect technique; **MRR**= Mark–release–recapture; **NA**= Not available

**Supplementary References**

1. FAO, IAEA: **Thematic Plan for the Development and Application of the Sterile Insect Technique (SIT) and Related Genetic and Biological Control Methods for Disease Transmitting Mosquitoes**. In*.*; 2019: 94.

2. Velo E, Balestrino F, Kadriaj P, Carvalho D, Dicko AH, Bellini R, Puggioli A, Petric D, Michaelakis A, Schaffner F *et al*: **A Mark‑Release‑Recapture study to estimate field performance of imported radio-sterilized male *Aedes albopictus* in Albania**. *Frontiers in Bioengineering and Biotechnology* 2022, **10**:833698.

3. Hossain MF, Ghosh A, Sultana N, Momen M, Hossain MA, Khan SA, Seheli K: **Optimization of irradiation sterility dose of the male *Aedes aegypti* (Linnaeus) Mosquito: A laboratory study in Bangladesh**. *Int J Trop Insect Science* 2022, **42**(2):1421-1428.

4. Bouyer J, Culbert N, Dicko AH, Klaptocz A, Germann J, Wallner T, Herranz GS, Argiles Herrero R, Virgilio J, Gomez M *et al*: **Field performance of sterile male mosquitoes released from an uncrewed aerial vehicle**. *Science Robotics* 2020, **5**(43):eaba6251.

5. Virginio JF: **Country report on the ongoing pilot SIT trials and plans for epidemiological trials in Brazil**. *Infectious Diseases of Poverty* 2024, **Special Issue on TDR Training Workshop on SIT (Tahiti, May 2023)**:submitted.

6. Zhang D, Bouyer J: **Mating harassment may boost the effectiveness of the sterile insect technique for *Aedes* mosquitoes**. *Nature communications* 2024, **in press**.

7. Zheng X, Zhang D, Li Y, Yang C, Wu Y, Liang X, Yan Z, Hu L, Sun Q, Liang Y *et al*: **Incompatible and sterile insect techniques combined eliminate mosquitoes**. *Nature* 2019, **572**:56-61.

8. Gato R, Menéndez Z, Prieto E, Argilés R, Rodríguez M, Baldoquín W, Hernández Y, Pérez D, Anaya J, Fuentes I *et al*: **Sterile Insect Technique: Successful Suppression of an *Aedes aegypti* Field Population in Cuba**. *Insects* 2021, **12**(5):469.

9. Gato R: **SIT in Cuba**. *Infectious Diseases of Poverty* 2024, **Special Issue on TDR Training Workshop on SIT (Tahiti, May 2023)**:submitted.

10. Vasquez MI, Notarides G, Meletiou S, Patsoula E, Kavran M, Michaelakis A, Bellini R, Toumazi T, Bouyer J, Petrić D: **Two invasions at once: update on the introduction of the invasive species Aedes aegypti and Aedes albopictus in Cyprus–a call for action in Europe**. *Parasite* 2023, **30**:41.

11. Bossin H: **SIT in the Pacific**. *Infectious Diseases of Poverty* 2024, **Special Issue on TDR Training Workshop on SIT (Tahiti, May 2023)**:submitted.

12. Gouagna LC, Damiens D, Oliva CF, Boyer S, Le Goff G, Brengues C, Dehecq J-S, Raude J, Simard F, Fontenille D: **Strategic approach, advances, and challenges in the development and application of the SIT for area-wide control of Aedes albopictus mosquitoes in Reunion Island**. *Insects* 2020, **11**(11):770.

13. Simard F: **SIT in Indian Ocean**. *Infectious Diseases of Poverty* 2024, **Special Issue on TDR Training Workshop on SIT (Tahiti, May 2023)**:submitted.

14. Bouyer J, Almenar D, Tur C, Pla Mora I, Hamidou M, Mamai W, Yamada H, Gouagna LC, Rossignol M, Chandre F *et al*: **Suppression of *Aedes* mosquito populations using boosted sterile insect technique in contrasted environments**. *Scientific Reports* 2024, **in press**.

15. Becker N, Langentepe-Kong SM, Tokatlian Rodriguez A, Oo TT, Reichle D, Lühken R, Schmidt-Chanasit J, Lüthy P, Puggioli A, Bellini R: **Integrated control of *Aedes albopictus* in Southwest Germany supported by the Sterile Insect Technique**. *Parasites & Vectors* 2022, **15**(1):1-19.

16. Balatsos G, Karras V, Puggioli A, Balestrino F, Bellini R, Papachristos DP, Milonas PG, Papadopoulos NT, Malfacini M, Carrieri M *et al*: **Sterile Insect Technique (SIT) field trial targeting the suppression of *Aedes albopictus* (Skuse) in Greece**. *Parasite* 2024, **31**(17):11.

17. Sasmita HI: **Country report on the ongoing pilot SIT trials and plans for epidemiological trials in Indonesia**. *Infectious Diseases of Poverty* 2024, **Special Issue on TDR Training Workshop on SIT (Tahiti, May 2023)**:submitted.

18. Bellini R, Medici A, Puggioli A, Balestrino F, Carrieri M: **Pilot field trials with *Aedes albopictus* irradiated sterile males in Italian urban areas**. *J Med Entomol* 2013, **50**(2):317-325.

19. Bouyer J, Pla Mora I, Mikaelakis A, Bellini R: **New developments in the use of the Sterile Insect Technique against *Aedes albopictus* in Europe**. In: *5th International Workshop on Aedes albopictus, the Asian tiger mosquito* vol. Invited conference. Montpellier: IRD; 2022: 23.

20. Nazni WA, Teoh G-N, Shaikh Norman Hakimi SI, Muhammad Arif MA, Tanusshni M, Nuradila MA, Nurfarahin Hanini A, Shazia IA, Tan A-M, Rabizah H: ***Aedes* Control Using Sterile Insect Technique (SIT) in Malaysia**. In: *Genetically Modified other Innovative Vector Control Technologies: Eco-bio-social Considerations for Safe Application.* edn. Edited by Tyagi BK. Singapore: Springer; 2021: 143-162.

21. Ling CY: **Country report on the ongoing pilot SIT trials and plans for epidemiological trials in Malaysia**. *Infectious Diseases of Poverty* 2024, **Special Issue on TDR Training Workshop on SIT (Tahiti, May 2023)**:submitted.

22. Iyaloo DP, Bouyer J, Facknath S, Bheecarry A: **Pilot Suppression trial of *Aedes albopictus* mosquitoes through an Integrated Vector Management strategy including the Sterile Insect Technique in Mauritius**. *bioRxiv* 2020, [**https://doi.org/10.1101/2020.09.06.284968**](https://doi.org/10.1101/2020.09.06.284968).

23. Marina CF, Liedo P, Bond JG, R. Osorio A, Valle J, Angulo-Kladt R, Gómez-Simuta Y, Fernández-Salas I, Dor A, Williams T: **Comparison of ground release and drone-mediated aerial release of *Aedes aegypti* sterile males in southern Mexico: efficacy and challenges**. *Insects* 2022, **13**(4):347.

24. Marina CF, Bond JG, Hernández-Arriaga K, Valle J, Ulloa A, Fernández-Salas I, Carvalho DO, Bourtzis K, Dor A, Williams T: **Population dynamics of *Aedes aegypti* and *Aedes albopictus* in two rural villages in southern Mexico: Baseline data for an evaluation of the sterile insect technique**. *Insects* 2021, **12**(1):58.

25. Martín-Park A, Che-Mendoza A, Contreras-Perera Y, Pérez-Carrillo S, Puerta-Guardo H, Villegas-Chim J, Guillermo-May G, Medina-Barreiro A, Delfín-González H, Méndez-Vales R *et al*: **Pilot trial using mass field-releases of sterile males produced with the incompatible and sterile insect techniques as part of integrated *Aedes aegypti* control in Mexico**. *PloS Negl Trop Dis* 2022, **16**(4):e0010324.

26. Lees RS, Carvalho DO, Bouyer J: **Potential impact of integrating the sterile insect technique into the fight against disease-transmitting mosquitoes**. In: *Sterile Insect Technique Principles and Practice in Area-Wide Integrated Pest Management.* Second edition edn. Edited by Dyck AV, Hendrichs J, Robinson AS. Vienna: CRC Press; 2021: 1082-1118.

27. Maiga H, Bakhoum MT, Mamai W, Diouf G, Somda NSB, Wallner T, Martina C, Kotla SS, Masso OB, Yamada H *et al*: **From the lab to the field: Long-distance transport of sterile *Aedes* mosquitoes**. *Insects* 2023, **14**(2):207.

28. Ng LC: ***Wolbachia*-mediated sterility suppresses *Aedes aegypti* populations in the urban tropics**. *medRxiv* 2021, **10.1101/2021.06.16.21257922**

29. Lim JT, Bansal S, Chong CS, Dickens BS, Ng Y, Deng L, Lee CW, Tan LW, Chain G, Ma P *et al*: **Efficacy of *Wolbachia*-mediated sterility to reduce the incidence of dengue: a synthetic control study in Singapore**. *The Lancet Microbe* 2024, **5**(5):e422-e432.

30. Kaiser ML, Wood OR, Damiens D, Brooke BD, Koekemoer LL, Munhenga G: **Estimates of the population size and dispersal range of *Anopheles arabiensis* in Northern KwaZulu-Natal, South Africa: implications for a planned pilot programme to release sterile male mosquitoes**. *Parasites & Vectors* 2021, **14**(1):1-18.

31. Tur C, Almenar D, Zacarés M, Benlloch-Navarro S, Pla I, Dalmau V: **Suppression Trial through an Integrated Vector Management of Aedes albopictus (Skuse) Based on the Sterile Insect Technique in a Non-Isolated Area in Spain**. *Insects* 2023, **14**(8):688.

32. Tur C, Almenar D, Benlloch-Navarro S, Argilés-Herrero R, Zacarés M, Dalmau V, Pla I: **Sterile insect technique in an integrated vector management program against tiger mosquito Aedes albopictus in the Valencia region (Spain): operating procedures and quality control parameters**. *Insects* 2021, **12**(3):272.

33. Dheerasinghe AF: **Country report on the ongoing pilot SIT trials and plans for epidemiological trials in Sri Lanka**. *Infectious Diseases of Poverty* 2024, **Special Issue on TDR Training Workshop on SIT (Tahiti, May 2023)**:submitted.

34. Ageep TB, Damiens D, Alsharif B, Ahmed AB, Salih EHO, Ahmed FTA, Diabaté A, Lees RS, Gilles JRL, El Sayed B: **Participation of irradiated *Anopheles arabiensis* males in swarms following field release in Sudan**. *Malar J* 2014, **13**(1):1-11.

35. Elaagip A, Adedapo A: **Three Decades of Malaria Vector Control in Sudan: The Plausible Role of Sterile Insect Technique (SIT)**. In: *Genetically Modified other Innovative Vector Control Technologies: Eco-bio-social Considerations for Safe Application.* edn. Edited by Tyagi BK; 2021: 119-129.

36. Flacio E: **SIT in Switzerland against *Aedes albopictus***. *Infectious Diseases of Poverty* 2024, **Special Issue on TDR Training Workshop on SIT (Tahiti, May 2023)**:submitted.

37. Kittayapong P, Ninphanomchai S, Limohpasmanee W, Chansang C, Chansang U, Mongkalangoon P: **Combined sterile insect technique and incompatible insect technique: The first proof-of-concept to suppress Aedes aegypti vector populations in semi-rural settings in Thailand**. *PloS Negl Trop Dis* 2019, **13**(10):e0007771.

38. Foley N: **SIT in Florida**. *Infectious Diseases of Poverty* 2024, **Special Issue on TDR Training Workshop on SIT (Tahiti, May 2023)**:submitted.

39. Chen C, Aldridge RL, Gibson S, Kline J, Aryaprema V, Qualls W, Xue Rd, Boardman L, Linthicum KJ, Hahn DA: **Developing the radiation‐based sterile insect technique (SIT) for controlling *Aedes aegypti*: identification of a sterilizing dose**. *Pest manag sci* 2023, **79**(3):1175-1183.
